# Systematic Review of Femoral Artery Stent Fractures

**DOI:** 10.1016/j.ejvsvf.2024.08.001

**Published:** 2024-08-17

**Authors:** Arielle Bellissard, Salomé Kuntz, Anne Lejay, Nabil Chakfé

**Affiliations:** a Department of Vascular Surgery, Kidney Transplantation and Innovation, Strasbourg University Hospital, Strasbourg, France; b GEPROMED, Bâtiment d’Anesthésiologie, Strasbourg, France

**Keywords:** Femoropopliteal artery, Peripheral arterial disease Stent fracture

## Abstract

**Objective:**

Primary stenting for long femoropopliteal (FP) lesions remains controversial because of the high risk of stent fracture (SF). This study aimed to summarise current knowledge on SF from randomised control trials about FP stenting.

**Methods:**

A systematic review of the Medline database was performed by a combined strategy of MeSH terms: femoral artery, popliteal artery, stenting, and stent fracture. SF was classified according to a standard classification: 1 = single strut fracture; 2 = ≥ two struts fracture; 3 = type 2 with deformation; 4 = multiple struts fracture with acquired transection; 5, type 4 with gap in the stent body.

**Results:**

The literature search identified 25 publications including covered stents (CSs; *n* = 3), drug eluting stents (DESs; *n* = 8), bare metal stents (BMS; *n* = 17), and bioabsorbable stents (*n* = 1). Data were extracted from 4 047 patients; mean age ± standard deviation was 68.9 ± 3.0 years and 69% were male. The median lesion length was 87.6 mm (interquartile range [IQR] 70.0, 149) with a median chronic total occlusion proportion of 36.8% (IQR 29.0, 56.5). In 208 patients treated with CS, SF rates ranged from none to 2.6% at 36 months with no clinical correlation. In 1 106 patients treated with DES, SF rates were relatively low in large cohorts, ranging from 0% at 12 months to 1.9% at 60 months. In smaller cohorts (under 100 patients per group), they ranged from 12.5% at six months to 46.7% at 12 months, with no clinical repercussion. In 1 610 patients treated with BMS, SF rates ranged from 2% to 32.7% at 12 months and from 2.9% to 48.9% at 24 months, with no clinical repercussion.

**Conclusion:**

SF rates in large cohorts were low in CF and DES, and quite common in BMS, although none of them had clinical consequences. However, longer follow up and detailed, accurate reports are needed to assess eventual real clinical outcomes.

## Highlights

• Stent fracture rates in femoropopliteal arteries are low and decrease over time.
• Stent fracture rates are low with covered stents and drug eluting stents.
• Stent fracture rates are more frequent with bare metal stents.
• Stent fractures have little clinical relevance in up to three year follow up trials.

## INTRODUCTION

With an ageing population, endovascular interventions are nowadays considered the first line strategy for the treatment of peripheral arterial obstructive disease.^1^ However, achieving long term patency remains a challenge, especially in the femoropopliteal (FP) artery.^2^ Technical success does not seem to be altered, with successful recanalisation rates of TransAtlantic Inter-Society Consensus C and D lesions exceeding 80%, but endovascular treatment of long FP lesions requires continuous surveillance and long follow up.^3^

Discussions are ongoing on whether FP treatment should be restricted to percutaneous transluminal angioplasty (PTA) or is best achieved with stenting.^4^ Stenting may improve patency rates, but remains controversial in longer FP lesions because of the high risk of stent fracture (SF).^5^^,^^6^ Indeed, compared with other peripheral arteries, the FP segment is subjected to high mechanical stress with more axial torsion and longitudinal deformation that could ultimately lead to more SF.^7^^,^^8^ Other factors seems to favour SF in the FP segment, such as intensive walking.^9^

Thus, the aim of this study was to discuss SF rates and repercussion in FP arteries, with a systematic literature review of available data from randomised control trials (RCTs) about FP stenting.

## MATERIALS AND METHODS

**Figure 1:**
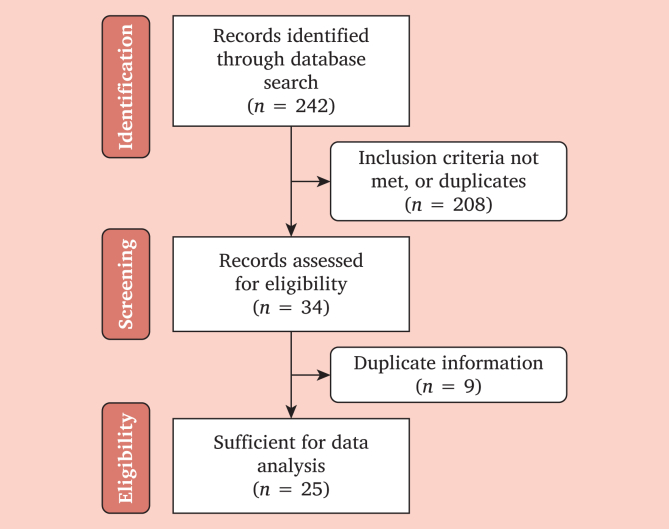
Flowchart of the systematic review. The systematic literature review was performed following Preferred Reporting Items for Systematic reviews and Meta-Analyses guidelines.^10^

The systematic literature review was performed following the Preferred Reporting Items for Systematic reviews and Meta-Analyses guidelines (Fig. 1).^10^

### Eligibility criteria

Studies included were full text English language publications without any chronological limit. All RCTs reporting on SF and including stenting in the FP segment (defined as the common femoral artery [CFA], superficial femoral artery [SFA], and popliteal artery) in at least one of their groups were included, with no regards for indication of stenting or additional surgical procedure. Exclusion criteria were study type other than RCT and absence of SF mention in the RCT result section. Same studies at different time points were also excluded, in order to not duplicate the data. The main outcome of interest was the presence of SFs and their clinical implications.

### Information sources and search strategy

The Medline and Embase databases were searched with a combined strategy using the terms femoral artery, popliteal artery, stenting, stent fracture. Two investigators (S.K., A.B.) screened all titles and abstracts collected for relevance. When a relevant article was found, full text articles were reviewed. Studies that did not meet the inclusion criteria were excluded. The reference lists of included studies were searched, and the first 20 related items in Medline were scanned for relevant studies.

### Study records and data items

Extracted data included year of publication, study design, patient and lesion characteristics, device type, SF diagnosis, SF type and rate, and clinical outcomes associated with SF. SFs were classified as follows, according to a coronary arteries classification^11^ that was adapted for peripheral arteries: type 1 = single strut fracture; type 2 = two or more struts fracture without deformation; type 3 = two or more struts fracture with deformation; type 4 = multiple struts fracture with acquired transection but without gap; and type 5 = multiple struts fracture with acquired transection with gap in the stent body.

### Statistical analysis

Results for continuous variables with normal distribution were expressed as mean ± standard deviation (SD). Variables with non-normal distribution were expressed as median and interquartile range (IQR). Normality of distribution was tested using the Shapiro–Wilk test.

## RESULTS

Fig. 1 shows the selection process. Data extraction led to the evaluation of 242 publications, of which 34 publications met the inclusion criteria. Nine studies were excluded due to duplicate information. A total of 25 publications including covered stents (CSs; *n* = 3),^12^, ^13^, ^14^ drug eluting stents (DESs; *n* = 8),^15^, ^16^, ^17^, ^18^, ^19^, ^20^, ^21^, ^22^ bare metal stents (BMSs; *n* = 17),^13^^,^^15^, ^16^, ^17^^,^^21^^,^^23^, ^24^, ^25^, ^26^, ^27^, ^28^, ^29^, ^30^, ^31^, ^32^, ^33^, ^34^^,^^36^ and bioabsorbable stents (*n* = 1)^35^ were analysed. Studies addressed SFs in the FP segment in most cases; one study addressed SFs in the popliteal segment^29^ and two in the CFA.^35^^,^^36^

Data were extracted from 4 047 patients; mean age ± SD was 68.9 ± 3.0 years and 69% were male patients. Median lesion length was 87.6 mm (IQR 70.0, 149) with a median chronic total occlusion (CTO) proportion of 36.8% (IQR 29.0, 56.5). SF was diagnosed with angiography in 23% of stented patients, with plain Xray in 59% and with ultrasound in 15%.

The paucity of the available data did not enable a proper meta-analysis.

### Stent fracture with covered stent in the femoropopliteal segment

Publications reporting on SF in CS (*n* = 3) addressed FP stenting with the Viabahn Endoprosthesis (WL Gore & Associates, Flagstaff, USA) (Table 1).^12^, ^13^, ^14^ There was no report on other type of CS. A total of 428 patients were enrolled, including 208 patients with CSs. The mean age ± SD was 67.3 ± 1.8 years and 71% were male. The median lesion length treated was 176.5 mm (IQR 70.0, 190.0) with a median of 27% CTO (IQR 22.5, 57.7). SF rates in CSs were low and ranged from none to 2.6% at 36 months.

**Table 1:** Studies including stent fractures (SFs) with covered stents in the femoropopliteal artery.

Publication	RCT design	Patients – *n*	Indication (Rutherford)	Stent name	Mean lesion length – mm	CTO –%	Stent fracture	SF type	SF per subject and time point	Outcome
Saxon^12^ (2008)	CS *vs.* PTA	97	2–6	Viabahn	70	20	No			
Gerarghty^13^ (2013)	CS *vs.* BMS	72	1–5	Viabahn	190	61	Yes	Type 1	2.1% at 12 mo, 4.7% at 24 mo, 2.6% at 36 mo	None
		76		Nitinol stent, unspecified model	180	57	Yes	Type 1–5	32.7% at 12 mo, 48.9% at 24 mo, 50% at 36 mo	None
Bosiers^14^ (2020)	CS *vs.* PTA	39	2–5	Viabahn	173	23	No			

BMS = bare-metal stent; CS = covered stent; CTO = chronic total occlusion; NA = not available; PTA = percutaneous transluminal angioplasty; RCT = randomised control trial; SF = stent fracture.

Saxon *et al*. compared the treatment of SFA lesions of 13 cm or shorter with PTA alone *vs.* PTA followed by Viabahn placement. No SF was identified at 12 months.^12^ Geraghty *et al*. reported the results of CS with the Viabahn *vs.* nitinol BMS for complex SFA lesions. SFs were significantly more common in BMS (50.0%) than in the Viabahn (2.6%) and associated with longer treated segments. Fracture severity was significantly greater for BMS: type 1 and 2 fractures for Viabahn *vs*. all types of fractures including 3 and 4 for BMS. SFs did not correlate with bad outcomes.^13^ Bosiers *et al*. compared the outcome of Viabahn with standard PTA for the treatment of in stent re-stenosis in the FP arteries. No SFs were observed throughout 12 months.^14^

### Stent fracture with drug eluting stent in the femoropopliteal segment

Publications addressing fractures in DESs in the FP artery (*n* = 8) reported on three types of stents: two paclitaxel coated stents (Zilver PTX, Cook Medical, Bloomington, IN, USA and Eluvia, Boston Scientific, Marlborough, MA, USA), and one sirolimus coated stent (Smart, Cordis, Miami Lake, FL, USA) (Table 2).^15^, ^16^, ^17^, ^18^, ^19^, ^20^, ^21^, ^22^ A total of 1 539 patients were enrolled in these studies, including 1 106 patients with DES, with a median age of 67.7 years (IQR 65.9, 69.3); 79% were male. The median lesion length was 82.4 mm (IQR 71.5, 202.9) with a median of 56.7% (IQR 32.4, 93.3) CTOs. SFs in DESs in large cohorts ranged from 0% at 12 months to 1.9% at 60 months. In smaller cohorts (studying fewer than 100 patients per stent group), SFs ranged from 12.5% at six months to 46.7% at 12 months.

**Table 2:** Studies including stent fractures (SFs) with drug eluting stents in the femoropopliteal artery.

Publication	RCT design	Patients – *n*	Indication (Rutherford)	Stent name	Mean lesion length – mm	CTO – %	Stent fracture	SF type	SF per subject and time point	Outcome
Duda^15^ (2002)	DES *vs.* BMS	18	1–4	Smart + sirolimus	83	89	Yes	NA	16.7% at six mo	None
		18		Smart	56	59	Yes	NA	16.7% at six mo	None
Duda^16^ (2006)	DES *vs.* BMS	47	1–4	Smart + sirolimus	85	69	Yes	NA	36% at 18 mo	1 vessel ulceration with covered stent placement
		47		Smart	81	57	Yes	NA	20% at 18 mo	
Dake^17^ (2016)	DES *vs.* PTA	236	2–6	Zilver PTX	66	33	Yes	Type 1 and 3	0.9% at 12 mo, 1.9% at 60 mo	None
		69		Zilver PTX	63	27	No			
		51		Zilver	63	27	No			
Mikki^18^ (2017)	DES 6 mm *vs.* 8 mm	10	2–6	Zilver PTX	70	NA	Yes	Type 1	12.5% at six mo	None
		10		Zilver PTX	78	NA	No			
Gray^19^ (2018)	DES *vs.* DES	309	2–4	Eluvia	87	31	Yes	Type 3	0.3% at 12 mo	None
		156		Zilver PTX	82	30	No			
Bosiers^20^ (2020)	DES *vs.* Bypass	113	2–5	Zilver PTX	241	92	No			
Gouëffic^21^ (2020)	DES *vs.* BMS	85	2–5	Misago	76	35	Yes	Type 3 and 4	2.9% at 24 mo	None
		86		Zilver PTX	69	38	Yes	Type 4	1.3% at 24 mo	None
Cheban^22^ (2023)	DES + Hunter fasciotomy *vs.* DES alone	30	4–6	Zilver PTX	249	100	Yes	Type 1–5	23.3% at 12 mo	Correlated with re-stenosis
		30		Zilver PTX	260	100	Yes	Type 1–5	46.7% at 12 mo	Correlated with re-stenosis

BMS = bare metal stent; CTO = chronic total occlusion; DES = drug eluting stent; NA = not available; PTA = percutaneous transluminal angioplasty; RCT = randomised control trial; SF = stent fracture.

Studies involving the Zilver PTX stents reported a SF rate ranging from 0 at 12 months to 1.9% at 60 months.^17^, ^18^, ^19^, ^20^, ^21^, ^22^ The study with the highest SF rate reported 46.7% SFs at 12 months and correlated SF with re-stenosis.^22^ In the other studies, none of the SFs were associated with a pejorative clinical outcome.

One study involved the Eluvia stent and reported a SF rate of 0.3% at 12 months. Only one of 319 implanted Eluvia stents had grade 3 SF. The vessel was patent, and there were no major adverse events at 12 months.^19^

Two studies involved sirolimus coated Smart stents.^15^^,^^16^ The first study compared BMS and sirolimus stents and reported a 16.7% SF rate at six months. The SF rate was similar between the two groups and SFs occurred where three stents were overlapping.^15^ In the second study, the SF rate was 36% at 18 months. All patients with SFs were clinically asymptomatic, but one patient underwent prophylactic CS placement over a 12 mm vessel ulceration at the site of the fracture.^16^

### Stent fracture with bare metal stent in the femoropopliteal segment

Publications addressing BMS fractures in the FP artery (*n* = 16) included eight types of stents: Absolute (Abbott Vascular, Abbott Park, IL, USA), LifeStent and Luminexx (Becton, Dickinson and Company, Franklin Lake, NJ, USA), Wallstent (Boston Scientific), Smart (Cordis), Dynalink (Guidant Corporation, Saint Paul, MN, USA), Misago (Terumo, Tokyo, Japan), and Tigris (WL Gore & Associates), which is no longer manufactured (Table 1, Table 2, Table 3).^13^^,^^15^, ^16^, ^17^^,^^21^^,^^23^, ^24^, ^25^, ^26^, ^27^, ^28^, ^29^, ^30^, ^31^, ^32^, ^33^, ^34^ A total of 2 261 patients were enrolled in these studies, including 1 610 patients with BMSs, with a mean ± SD age of 69.0 ± 3.0 years; 69% were male. The median lesion length was 90.3 mm (IQR 69.4, 129.9) with a median of 38.5% (IQR 32.3, 55.0) CTOs. SF rates in BMSs ranged from 2% to 32.7% at 12 months and from 2.9% to 48.9% at 24 months. SFs were not correlated with clinical outcomes.

**Table 3:** Studies including stent fractures (SFs) with bare metal stents in the femoropopliteal artery.

Publication	RCT design	Patients – *n*	Indication (Rutherford)	Stent name	Mean lesion length – mm	CTO – %	Stent fracture	SF type	SF per subject and time point	Outcome
Schillinger^23^ (2006)	BMS *vs.* PTA	51	3–5	Dynalink, Absolute	101	37	Yes	NA	2% at six mo, 2% at 12 mo	NA
Krankenberg^24^ (2007)	BMS *vs.* PTA	123	2–6	Luminexx	45	37	Yes	NA	12% at 12 mo	None
Iida^25^ (2008)	BMS + Cilostazol *vs.* BMS + Ticlopidine	56	2–6	Luminexx, Wallstent	141	51	Yes	NA	19.6% at 12 mo	NA
		54		Luminexx, Wallstent	135	43	Yes	NA	16.7% at 12 mo	NA
Soga^26^ (2009)	BMS + Cilostazol *vs.* BMS alone	16	1–3	Luminexx, Wallstent, Smart	121	26	Yes	NA	6.3% at 24 mo	No re-stenosis at fracture site
		20		Luminexx, Wallstent, Smart	132	36	Yes	NA	10% at 24 mo	No re-stenosis at fracture site
Laird^27^ (2012)	BMS *vs.* PTA	134	1–3	LifeStent	71	17	Yes	Type 1 and 4	0.3% at six mo, 3.1% at 12 mo, 4.1% at 18 mo	None
Iida^28^ (2013)	BMS + Cilostazol *vs.* BMS alone	82	2–4	Smart	132	40	Yes	NA	17% at 12 mo	None
		88		Smart	125	39	Yes	NA	20% at 12 mo	None
Rastan^29^ (2015)	BMS *vs.* PTA	119	2–5	LifeStent	41	NA	Yes	Type 1–5	4.6% at 24 mo	None
Therasse^30^ (2016)	BMS + external beam therapy *vs.* BMS alone	78	1–6	Smart	60	31	Yes	Type 1, 2 and 3	8.5% at 24 mo	NA
		77		Smart	73	33	Yes	Type 1, 2 and 3	15.1% at 24 mo	NA
Laird^31^ (2018)	BMS *vs.* BMS	197	2–4	Tigris	108	42	No			
		70		LifeStent	118	37	Yes	Type 1–5	27.1% at 12 mo, 32.7% at 24 mo	None
Iida^32^ (2019)	BMS *vs.* PTA	51	1–3	Smart	35	29	Yes	Type 1	2% at 36 mo	None
Ko^33^ (2019)	Spot *vs.* long stenting	59	1–5	Smart	245	90	Yes	Type 1 and 2	8% at 12 mo	None
		66		Smart	238	88	Yes	Type 1, 2 and 3	8.3% at 12 mo	None
Kato^34^ (2022)	BMS + intensive *vs.* non-intensive exercise	22	2–4	Nitinol stent, unspecified model	100	50	No			
		22		Nitinol stent, unspecified model	65	43	Yes	NA	9.5% at 12 mo	None

BMS = bare metal stent; CTO = chronic total occlusion; NA = not available; PTA = percutaneous transluminal angioplasty; RCT = randomised control trial; SF = stent fracture.

Seven studies included Smart stents. The SF rate ranged from 8% at 12 months to 20% at 24 months, with no clinical repercussions.^15^^,^^16^^,^^26^^,^^28^^,^^30^^,^^32^^,^^33^ One study used Smart stents along with Luminex and Wallstent and reported an altogether SF rate of 8.3% at 12 months. No re-stenosis occurred at the fracture site.^26^ Another study used both the Luminex and Wallstent stents and reported a total SF rate of 18.1% at 12 months.^25^ One study compared the Luminexx stent with PTA in short FP lesions. SF rates were 12% at 12 months. The binary re-stenosis rate in patients with SF was not statistically different from patients without SF (20.0 % *vs.* 28.8 %, respectively; *p* = 0.72).^24^

Three studies included LifeStent stents.^27^^,^^29^^,^^31^ They reported SFs ranging from 3.1% to 27.1% at 12 months and 4.6%–32.7% at 24 months. The first study compared PTA with stenting in intermediate FP lesions (<150 mm). At 18 months, they reported 4.1% of SFs: type 1 (five stents), type 4 (six stents), or both (one stent). A *post hoc* subset analysis of patients with SF *vs.* patients with stent integrity demonstrated no relationship between SFs and clinical outcome. The freedom from target lesion revascularisation in the SF group at 36 months was estimated by Kaplan–Meier analysis at 90%.^27^ The second study also compared LifeStent to PTA but exclusively in popliteal lesions. At 24 months, 4.6% stents demonstrated SFs. No correlation was found between SF and re-stenosis or target lesion revascularisation.^29^ The third study compared LifeStent with Tigris for the treatment of intermediate lesions in the FP segment. There were no SFs in the Tigris group at 12 and 24 months, whereas the LifeStent group had a significantly higher SF rate of 32.7 % at 24 months (*p* < 0.001), with a majority of type 4 and 5 SFs.^31^

Schillinger *et al*. compared primary stenting with self expanding nitinol stents (Dynalink or Absolute) with PTA and optional stenting for the treatment of SFA obstructions at 24 months. They reported a SF rate of 2% at six months and 12 months. The two year results showed no new SFs.^23^ Gouëffic *et al*. reported a Misago SF rate of 2.4 % at 24 months, with type 3 and 4 fractures but no clinical repercussions.^21^ Most recently, Kato *et al*. compared nitinol BMSs in FP lesions responsible of claudication, in intensive *vs.* non-intensive exercise training groups, with no precision on the stent model. The non-intensive group showed a SF rate of 9.5% at 12 months. No SFs were observed in the intensive group.^34^

### Stent fracture in the common femoral artery

In the CFA, Gouëffic *et al*. compared stainless steel BMSs with endarterectomy for *de novo* atherosclerotic lesions.^36^ A total of 117 patients, including 56 with stents, were included. Only one SF was observed at 24 months. The fracture was a type 3. No re-stenosis was observed, and no re-intervention was required.

One RCT by Linni *et al*. compared the implantation of a bioabsorbable Remedy stent (Kyoto Medical Planning Co, Kyoto, Japan) with surgery for lesions of the CFA.^35^ A total of 80 patients including 40 patients with stents were included. No SFs were identified.

## DISCUSSION

The main findings in this literature review on femoral stent RCTs were (1) SFs in CSs in the FP artery were very rare and did not lead to any clinical repercussions, (2) the SF rate in DESs was low, except for the sirolimus Smart stent, although very few led to clinical repercussions, and (3) SFs were quite common in BMSs, although few of them were linked to re-stenosis.

Although quite common in BMSs, the RCTs did not seem to find any clinical repercussion from SFs, in contrast to Scheinert *et al*., who reported a considerable risk of SF after long segment femoral artery stenting, associated with a higher in stent re-stenosis and re-occlusion rate.^6^ One potential explanation of the low rates of SF but also the rare clinical significance might be explained by the short follow up of these RCTs; only one of them included a follow up greater than 36 months, with a maximum of 60 months of follow up. Hence, the conclusions about impact of SF on stent patency from this study are limited, since clinical repercussions could happen later in the follow up. Indeed, a few case reports describe some late SF complications, such as pseudoaneurysm formation and re-stenosis.^37^, ^38^, ^39^, ^40^, ^41^ Stent perforation has also been described, leading to a large, compressive intramuscular haematoma with deep venous thrombosis.^42^ Moreover, the reported SF rates in these RCTs also seem to indicate a tendency for higher SF rates with longer follow up, supporting the need for long term analysis of SF incidence and clinical repercussions.

More recently, explanted femoral stents were investigated and three cases of SF were reported, only one resulting in thrombosis identified only by microcomputed tomography.^43^ This shows that conventional imaging used in clinical practice (plain Xray, ultrasound, and even computed tomography angiography) can fail to diagnose SF, which could mean that SF rates in this study were underestimated.

The differences in lesion length, CTO proportion, and disease severity can also explain the variation in SF incidence and could impact late clinical outcomes, since stenting of long FP lesions has been reported as a risk factor for in stent re-stenosis.^44^^,^^45^ Implantation of multiple stents can also be a predisposing factor for fractures,^46^ which has been described in some of these RCTs with SFs occurring at the point of stent overlap.^15^^,^^27^ The results also suggest a difference in SF rates between DESs and BMSs, with fewer SFs in DESs. However, when looking at RCTs comparing DES with BMS specifically, reported SF rates are even between the groups.^15^^,^^16^^,^^21^ All in all, this needs to be kept in mind when interpreting these results that the design of these RCTs did not include SF as a primary outcome, hence the lack of available data on SF type and clinical repercussions.

The particularity of the FP arterial segment is its exposure to special mechanical influences that may have a negative impact on vessel patency after endovascular treatment.^6^ The superficial course of the artery, with crossing of flexion points and interaction with the surrounding musculature, potentially exposes it to external forces, including compression, torsion, and elongation.^7^^,^^8^ Stent design might then play a role in the occurrence of SFs, with different designs adapting differently to these forces. The *in vitro* study from Nikanorov *et al*. showed that commercially available stents exhibit a variable ability to withstand chronic deformation in vitro, and their response is highly dependent on the type of deformation applied. Axial compression resulted in high rates of fracture in Luminexx (80%) and LifeStent FlexStar (50%); bending deformation resulted in high rates of fracture in Protégé EverFlex (100%), Smart Control (100%), and Luminexx (100%). When axial and bending deformation were combined, the Absolute had the lowest rate of fracture (3%).^47^ The primary mode of BMS patency loss was the ingrowth of intimal hyperplasia through the stent interstices, a process that appeared to be potentiated by nitinol SF in the mobile SFA territory.^6^ In an *ex* vivo study Müller-Hülsbeck *et al*. evaluated different nitinol stents intended for the SFA with mechanical fatigue tests. The seven stent types showed differences in the incidence of high strain zones, indicating a potential for SF. The Misago, LifeStent and Absolute stents presented no zones of high strain during bending tests; in the torsion test, the Smart stent also had no zones of high strain. The worst performing stent was Luminexx during all test cycles.^48^

When studying the popliteal segment specifically, SF rates did not differ from the FP segment as a whole and resulted in no clinical repercussions.^29^ Considering only RCTs were included, the SUPERB trial was not included, which reported long term outcomes of an interwoven nitinol stent (Supera, IDEV Technologies, Inc, Webster, MO, USA) in popliteal stenotic arterial disease, which achieved an excellent primary patency through 36 months.^49^ The intent of Supera was to design a stent flexible enough to mimic the reticular structure of native collagen and elastin within vessels, emphasising radial strength, flexibility, and kink resistance. SFs were distinctly uncommon with this stent, with a single facture event in the 36 month follow up period.

Concerning the CFA, the study from Gouëffic *et al*. reported a very low SF rate, even though concerns were raised for SF due to this high mobility region and further surgical approaches.^36^ A fractured CS in the CFA was also reported. The explant analysis revealed a torn cover and the angle formed by the struts corresponded with the membrane perforation; however, there were no clinical repercussions.^50^ Indeed, a recent study by Tijani *et al*. demonstrated that the CFA is a fixed segment during extension and flexion of the hip and stenting does not modify this observation.^51^

### Conclusion

SF rates are low after CS and DES placement in the FP segment, and more frequent with BMSs. SFs have no clinical repercussions in the majority of patients in up to three year follow up trials, although long term follow up is needed to assess the eventual mechanical fate of stents and related clinical outcomes. Stent design refinement might lead to fewer SFs in future.

## Funding

There was no funding source for this research and the authors report no conflict of interest.
